# Application-focused validation of a viability-selective PMA-qPCR assay for selected skin-associated and cosmetic-relevant bacteria

**DOI:** 10.3389/fmicb.2026.1873833

**Published:** 2026-08-11

**Authors:** Sejin Cheon, Jimin Yoo, Yunseok Oh, Haeun Lee, Dong-geol Lee, Seunghyun Kang, Chunho Park, Seyoung Mun, Young Mok Heo, Kyudong Han

**Affiliations:** 1 COSMAX BTI R&I Center, Bundang-Gu, Seongnam, Republic of Korea; 2 Department of Microbiology, College of Bio-convergence, Dankook University, Cheonan, Republic of Korea; 3 Center for Bio-Medical Engineering Core Facility, Dankook University, Cheonan, Republic of Korea; 4 Department of Cosmedical Materials, College of Bio-convergence, Dankook University, Cheonan, Republic of Korea

**Keywords:** *C*_q_–CFU, LOD, LOQ, PMA-qPCR, Staphylococcus epidermidis CICARIA

## Abstract

Accurate quantification of viable bacteria is essential for ensuring product safety and quality in the food, pharmaceutical, and cosmetics industries. However, conventional plate culture methods are time-consuming and fail to detect viable but non-culturable (VBNC) cells, which frequently occur under environmental stress or preservation conditions and can influence product stability and safety. Accordingly, the need for a quantification method that accounts for VBNC cells has increased. In this study, we optimized and validated a viability-selective quantification method by combining propidium monoazide (PMA), a membrane-impermeable dye, with quantitative real-time polymerase chain reaction (qPCR). *Staphylococcus aureus* BF00309, *Staphylococcus epidermidis* CICARIA, and *Lactobacillus jensenii* KCTC 5194 were analyzed to validate the method. PMA treatment effectively suppressed DNA amplification in dead cells, resulting in significantly higher *C*_q_ values than in untreated controls. Strong linear correlations between *C*_q_ values and colony-forming units (CFU) obtained by plate counting were observed for all strains (*R*^2^ > 0.99), enabling the establishment of a *C*_q_–CFU regression model for estimating viable cell numbers. The derived model successfully quantified membrane-intact viable cells, which may include potential VBNC-like populations, and showed high agreement with culture-based methods. Moreover, selective detection of target bacteria was achieved in mixed samples containing high concentrations of background microorganisms, with a stable detection limit of approximately 10^3^ CFU/mL. These results demonstrate that the optimized PMA-qPCR method provides a rapid complementary tool to conventional culture-based quantification and serves as a promising preliminary screening approach with strong potential for future applications in skin microbiome research and the evaluation of live bacteria-based products, pending further validation in complex product matrices.

## Introduction

Recent advances in microbial biotechnology have significantly expanded the potential applications of live bacteria across various industries, including functional foods, pharmaceuticals, and cosmetics (Arshad et al., 2025). In particular, accumulating evidence linking the balance of the skin microbiome to overall skin health has led to growing interest in probiotic-based cosmetic formulations (Suri et al., 2025). As these formulations move closer to commercialization, one of the most critical aspects of their development is the accurate quantification of viable bacteria (Davis, 2014). Inaccurate enumeration of live bacteria during product development poses substantial risks. Underestimation of bacterial counts may lead to sub-therapeutic or ineffective formulations, while overestimation could result in microbial overgrowth, compromising product stability and possibly causing adverse skin reactions (Dao et al., 2018). In addition, an experimental baseline is required to mitigate interference from complex formulation ingredients, such as surfactants or oils (Yossa et al., 2023). From both regulatory and functional perspectives, the precise and reliable quantification of viable cells is essential for ensuring product consistency, efficacy, and safety (Ahire et al., 2024). Traditionally, the quantification of live bacteria has relied on culture-based methods such as plate counting (Reasoner, 2004). While widely used, these methods are time-consuming and incapable of detecting viable but non-culturable (VBNC) cells, which are particularly prevalent in formulated or stressed environments (Baron et al., 2006; Fleischmann et al., 2021). Consequently, there is an increasing need for molecular-based techniques that are not only rapid and sensitive but also capable of detecting VBNC populations (Lee et al., 2026).

Quantitative real-time polymerase chain reaction (qPCR) has emerged as a powerful molecular tool for the specific and sensitive detection of bacterial DNA based on target gene amplification (Huang et al., 2009). However, conventional qPCR cannot distinguish between DNA derived from live and dead cells. To address this limitation, viability dyes such as PMAxx™, ethidium monoazide (EMA), and propidium monoazide (PMA) have been developed (Tekgül and Adıgüzel, 2025). These dyes selectively penetrate cells with compromised membranes (i.e., dead cells), where they intercalate into DNA. Upon light activation, they form covalent bonds with the DNA, rendering it non-amplifiable in PCR. Conversely, DNA from intact, viable cells remains unaffected and can be accurately quantified (Nocker et al., 2007). When combined with qPCR, this approach—commonly referred to as viability-qPCR (v-qPCR)—enables selective quantification of live bacteria with improved accuracy and operational efficiency compared to culture-based techniques (Dinu et al., 2024). Based on these experimental methods, quantitative analyses have been conducted on various bacterial species. Sedgley et al. performed quantitative analysis of Enterococcus to investigate antimicrobial resistance (Sedgley et al., 2005). Furthermore, in 2024, Guo et al. quantified Lactobacillus to expand the applications of probiotics (Guo J. et al., 2024). Collectively, these studies indicated that the scope of live bacteria quantification is steadily broadening, highlighting the need for further research on a wider range of bacteria strains.

Recently, as the number of products utilizing both probiotics and intentionally inactivated bacteria has increased rapidly in the cosmetics and food industries, accurately distinguishing and quantifying live and dead bacteria has emerged as a critical requirement for verifying product efficacy and ensuring quality control (Nye et al., 2023; Wu et al., 2025). In line with these industrial demands, optimizing a viability assay for skin-associated bacteria within cosmetic contexts poses unique challenges, particularly due to the lack of optimized protocols that can simultaneously distinguish closely related pathobionts and beneficial strains in complex, co-existing microbial states. To address these limitations, this study evaluates the validity of a target-specific PMA-qPCR method using an *in vitro* mixed-microbial background system containing three skin-associated bacteria. A primary focus is placed on *Staphylococcus epidermidis* CICARIA strain, a dominant member of the human skin microbiome known for its role in maintaining skin health, alongside *Staphylococcus aureus* and *Lactobacillus jensenii*. Rather than evaluating each species individually, this work analyzes the linearity, sensitivity, and reproducibility of the method under a mixed-DNA background system and compares the results with the conventional plate counting method. Consequently, this study provides a baseline molecular framework that serves as a promising preliminary screening approach prior to further validation in complex cosmetic formulation matrices (Puebla-Barragan and Reid, 2021).

## Materials and methods

### Preparing bacteria and cultivation

*Staphylococcus aureus* BF00309 and *Staphylococcus epidermidis* CICARIA were provided by the COSMAX Microbiome Bank (CMB), COSMAX BTI R&I Center, and *Lactobacillus* jensenii KCTC 5194 was obtained from the Korean Collection for Type Cultures (KCTC). *S. aureus* and *S. epidermidis* were cultured in Tryptic Soy Broth (TSB; MBcell, Korea) at 37°C with shaking at 180 rpm. *L. jensenii* was cultured in de Man, Rogosa and Sharpe (MRS; Difco, United States) broth at 37°C.

### Propidium monoazide treatment and bacterial genomic DNA extraction

For all experiments, bacterial cultures were adjusted to an optical density at 600 nm (*OD*_600_) of 1.0 using a NanoDrop One (ThermoFisher Scientific, United States) (Beal et al., 2020). The adjusted cultures were serially diluted 10-fold (10^0^ to 10^–9^) (Martini et al., 2024). For enumeration, 20 μL of each dilution was spread in triplicate onto agar plates and incubated at 37°C for 24–48 h. Colonies were counted on plates yielding 30–300 colonies to determine the Colony Forming Units (CFU) per mL. The initial concentrations of *S. aureus*, *S. epidermidis*, and *L. jensenii* were 5.22 × 10^8^, 3.22 × 10^8^, 2.02 × 10^8^ CFU/mL, respectively.

Samples were divided into four experimental groups to validate the PMA efficiency: Live cells with PMA (Live^*PMA*+^), Live cells without PMA (Live^*PMA*–^), Dead cells with PMA (Dead^*PMA*+^), and Dead cells without PMA (Dead^*PMA*–^) (Kendra et al., 2024). Live cells were prepared using the untreated culture, whereas dead cells were generated by autoclaving at 121°C (15 psi) for 20 min to ensure complete membrane disruption and establish a reliable control group (Wang G. et al., 2023; Wang J. et al., 2023). PMA treatment was performed using PMAxx™ Dye (Biotium, Fremont, CA, United States) at a final concentration of 50 μM for bacterial viability screening according to the manufacturer’s recommended protocol (Yasunaga et al., 2013; Zhu et al., 2018; Qin et al., 2023; Guo J. et al., 2024; Pedrolo et al., 2024; Kaur et al., 2025; Luo et al., 2025; Schwendener et al., 2025). The samples were incubated in the dark on ice for 15 min to allow dye penetration, followed by photo-activation for 15 min using a high-intensity visible light source (405 nm) to induce crosslinking (Ma et al., 2017; Lee et al., 2022). Immediately after treatment, genomic DNA was extracted from 500 μL of each sample using the QIAamp PowerFecal Pro DNA Kit (Qiagen, Germany) according to the manufacturer’s instructions. DNA was eluted in 40 μL of elution buffer.

### PMA-qPCR

qPCR was performed to quantify viable bacteria. The specific primers used are listed in Table 1. The primer sets targeting specific genomic regions (*greA* for *S. aureus* and a specific locus within plasmid CP093223.1 for *S. epidermidis*) were originally designed by our laboratory, and their unique sequence selectivities are officially protected under registered patents (Patent No. 10-2023-015029) (Kim et al., 2023). To evaluate the primer systems, additional specificity was supported through both empirical cross-amplification and *in silico* alignment analyses. Empirical PCR validation under cross-species genomic DNA backgrounds yielded clean, distinct single bands at the expected amplicon size (∼100 bp) without any non-specific amplification or primer-dimer formation (Supplementary Figure 1A). Furthermore, Sanger sequencing and subsequent NCBI BLASTN analysis of the amplified products confirmed specific alignment with the intended target species, supporting quantification reliability in complex microbial matrices (Supplementary Figures 1B,C). Primer information for each bacterium used in this study is as follows: forward primer 5′-CCAGGTGATGAAGAGGAAAGTT-3′, reverse primer 5′-CCATTAGGTAGTGGAACACGAA-3′ for *S. aureus*; forward primer 5′-AATAGCCAAGCATCAAATGCCAG-3′, reverse primer 5′-GGAATTATGTCCCAGGCCCTTT-3′ for *S. epidermidis*; forward primer 5′-GAATCTTGCTACCAGGCTTTG-3, reverse primer 5′-CTCAGAAACTCAGCTTTACAC-3′ for *L. jensenii* (Table 1).

**TABLE 1:** Information of primer sequence and annealing temperature.

Bacteria	Primer	Sequence (5′–3′)	Annealing temperature
*S. aureus*	Forward	CCAGGTGATGAAGAGGAAAGTT	60°C
	Reverse	CCATTAGGTAGTGGAACACGAA	
*S. epidermidis*	Forward	AATAGCCAAGCATCAAATGCCAG	60°C
	Reverse	GGAATTATGTCCCAGGCCCTTT	
*L. jensenii*	Forward	GAATCTTGCTACCAGGCTTTG	65°C
	Reverse	CTCAGAAACTCAGCTTTACAC	

qPCR was conducted using the CFX96 Touch Deep Well Real-Time PCR Detection System (Bio-Rad, United States) under the following conditions: initial denaturation at 95°C for 10 min, followed by 40 cycles of denaturation at 95°C for 10 s, annealing at the primer optimal annealing temperature for 15 s, and extension at 72°C for 15 s. Finally, a melting curve was performed at 95°C for 5 s, followed by a temperature increase from 65 to 95 in 0.5°C increments. The optimal annealing temperatures were 60°C for *S. aureus*, 65°C for *L. jensenii*, and 60°C for *S. epidermidis* (Table 1). The reactions mixture was performed in a final volume of 20 μL. Each reaction mixture consisted of 10 μL Taq Pro Universal SYBR qPCR Master Mix (Vazyme, China), 1 μL each of 10 μM forward and reverse primers, 1 μL template DNA, and 7 μL nuclease-free water.

### Standard curve construction

Standard curves were generated to correlate the cycle quantification (*C*_q_) values with the number of viable bacteria (Hwang et al., 2024; Upadhyay et al., 2025). The CFU per Reaction (CFU/Reaction) was calculated based on the plate count results (CFU/mL), taking into account the DNA extraction volume (500 μL), elution volume (40 μL), and the template volume used in qPCR (1 μL) (da Silva et al., 2023). The genomic DNA extracted from the serial dilutions (10^0^–10^–9^) corresponds to a range of 6.81 to −2.19 Log_10_ CFU/Reaction for *S. aureus*, 6.60 to −2.40 Log_10_ CFU/Reaction for *S. epidermidis*, and 6.40 to −2.60 Log_10_ CFU/Reaction for *L. jensenii*. qPCR assays were performed in triplicate using 1 μL of extracted DNA without further quantification steps.

To assess the precision and reproducibility of the assay, the coefficient of variation (CV) was calculated. Specifically, for the dilution range of 10^–4^ to 10^–6^, where colonies were within the countable range, 10 additional replicates of plate counting were performed. The CV was calculated for both the plate counts (CFU/mL) and the corresponding *C*_q_ values using the formula: CV (%) = (Standard Deviation/Mean) × 100. This analysis confirmed the reliability of the quantification within the linear dynamic range.

### Preparation of DNA mixtures for limit of detection analysis

To evaluate the limit of detection (LOD) of *S. aureus* under a background microbial matrix, genomic DNA mixtures mimicking a co-existing state were prepared (da Silva et al., 2023; Zhao et al., 2024). The background bacteria, *S. epidermidis* and *L. jensenii*, were adjusted to a concentration of 10^10^ CFU. The target bacteria, *S. aureus*, was prepared in a 10-fold serial dilution series ranging from 10^10^ to 10^0^ CFU. Cell pellets were obtained by centrifugation and subsequently treated with PMAxx™, followed by photo-activation using a high-intensity visible light source (405 nm) to inhibit DNA amplification from dead cells. Genomic DNA was then extracted individually from each strain using the QIAamp PowerFecal Pro DNA Kit. DNA was eluted in 30 μL of elution buffer. To simulate a mixed environment, the extracted DNA of *S. aureus* was mixed with that of the background bacteria (*S. epidermidis* or *L. jensenii*) in a 1:1 volume ratio. These DNA mixtures were subsequently analyzed by qPCR using species-specific primers.

In this study, the LOD and limit of quantification (LOQ) thresholds were evaluated based on the distinct microbiological and matrix characteristics of each strain, rather than relying on a singular conventional mathematical formula, aligning with recent validation trends for complex microbial assays (Zhao et al., 2024). Specifically, to systematically account for the cumulative stochastic variations and potential biological overdispersion originating from the initial cell dilution phases, a probabilistic framework was adapted for LOD characterization as outlined by Sharp et al. (2023), while the LOQ was established based on empirical precision and amplification consistency within the linear dynamic range (Zhao et al., 2024). This integrated approach allowed for a highly practical and robust estimation of target-specific detection boundaries under background DNA interference. Specifically, the reliable limit of detection (rLOD) was defined as the lowest target concentration at which positive amplification (*C*_q_ ≤ 40) was consistently achieved in at least 95% of the replicates. The limit of quantification (LOQ) was established based on empirical precision, using the lowest concentration that maintained an acceptable coefficient of variation (CV) of < 20% among the calculated quantities of technical replicates. Any inconsistent late-cycling signals appearing at *C*_q_ > 40 or failing to show reproducible log-linear amplification across replicates were filtered out and treated as undetected to prevent any quantitative biases.

### Statistics

Linear regression analysis was performed to determine the correlation between *C*_q_ and CFU/Reaction, and to calculate the coefficient of determination (*R*^2^) and amplification efficiency (*E*). All statistical analyses were conducted using GraphPad Prism v10.4 (GraphPad Software, Boston, MA, United States). A *P*-value of ≤ 0.05 was considered statistically significant.

## Results

### Validation of PMA-qPCR

**FIGURE 1:**
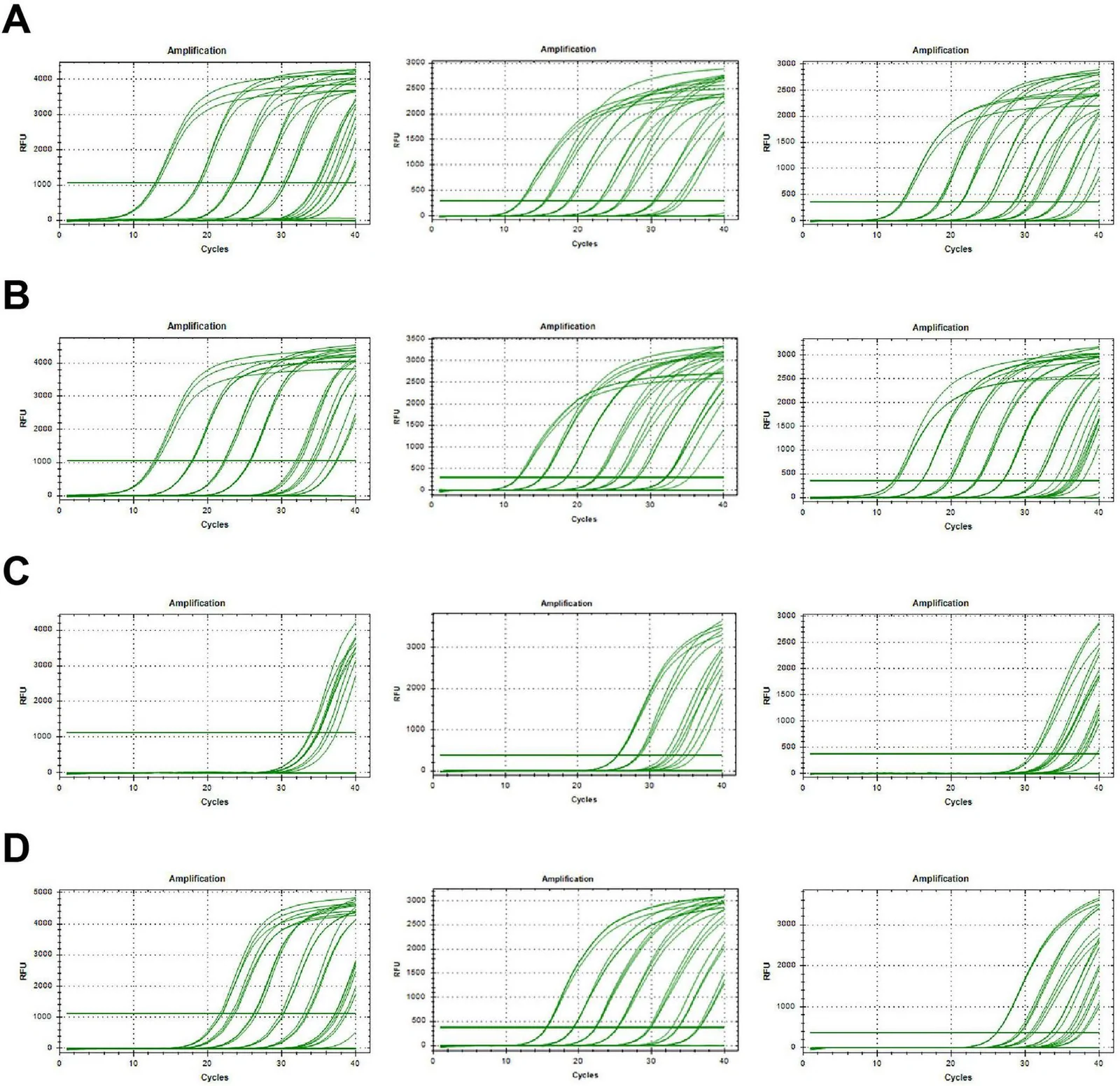
Evaluation of PMA performance through PMA treatment of Live and Dead Bacteria. qPCR amplification curves showing amplification dynamics across ten-fold serial dilutions (from 10^0^ to 10^– 9^). The left, center, and right panels represent the amplification curves for *S. aureus*, *S. epidermidis*, and *L. jensenii*, respectively. **(A)** Amplification curves of the Live^*PMA*+^ group. **(B)** Amplification curves of the Live^*PMA*–^ group. **(C)** Amplification curves of the Dead^*PMA*+^ group. **(D)** Amplification curves of the Dead^*PMA*–^ group.

To selectively quantify DNA derived from viable cells, excluding DNA derived from extracellular and dead cells, qPCR analysis was performed using PMA pretreatment. The effect of PMA on inhibiting DNA amplification of non-viable cells was clearly confirmed by comparing the qPCR results of Dead^*PMA*+^ and Dead^*PMA*–^ in all three bacteria (Figure 1). For Dead^*PMA*+^, qPCR results showed high *C*_q_ values (*S. aureus*: 34.60 ± 0.51–36.58 ± 0.70; *S. epidermidis*: 25.55 ± 0.07–32.54 ± 0.50; *L. jensenii*: 31.10 ± 0.24–34.30 ± 0.34) at dilutions of 10^0^, 10^–1^, and 10^–2^. In contrast, for *S. aureus*, signals fell below the reliable detection threshold (N/A) at dilutions of 10^–3^ and lower, though sporadic or inconsistent late amplification was still observed for *S. epidermidis* and *L. jensenii* at certain lower dilutions (10^–3^ and 10^–5^), as detailed in Table 2 (Figure 1C). On the other hand, Dead^*PMA*–^ exhibited relatively low *C*_q_ values (*S. aureus*: 23.42 ± 0.27–26.39 ± 0.24; *S. epidermidis*: 15.56 ± 0.08–22.31 ± 0.08; *L. jensenii*: 26.16 ± 0.02–30.87 ± 0.31) at the same dilutions (10^0^–10^–2^), and DNA amplification was observed even at dilutions of 10^–3^ and lower (Figure 1D). Furthermore, the effect of PMA was observed even in conditions where Live^*PMA*+^ and Live^*PMA*–^ (Figures 1A,B). As shown in Table 2, Live^*PMA*+^ consistently yielded slightly higher *C*_q_ values across all dilutions than Live^*PMA*–^.

**TABLE 2:** Quantification of PMA-qPCR and plate count method.

Target bacteria	Dilution	Plate counts			Average *C*_q_			
		Plate 1	Plate 2	Plate 3	Live^*PMA*+^	Live^*PMA*–^	Dead^*PMA*+^	Dead^*PMA*–^
*S. aureus*	10^0^	TNTC	TNTC	TNTC	13.03 ± 0.18	12.97 ± 0.21	34.60 ± 0.51	23.42 ± 0.27
*S. epidermidis*	10^0^	TNTC	TNTC	TNTC	12.23 ± 0.11	11.87 ± 0.12	25.55 ± 0.07	19.29 ± 0.06
*L. jensenii*	10^0^	TNTC	TNTC	TNTC	13.37 ± 0.14	12.39 ± 0.22	34.30 ± 0.34	30.87 ± 0.31
*S. aureus*	10^–1^	TNTC	TNTC	TNTC	18.83 ± 0.17	17.95 ± 0.09	34.61 ± 0.65	21.90 ± 0.20
*S. epidermidis*	10^–1^	TNTC	TNTC	TNTC	15.50 ± 0.16	15.08 ± 0.10	28.43 ± 0.17	15.56 ± 0.08
*L. jensenii*	10^–1^	TNTC	TNTC	TNTC	18.32 ± 0.14	16.02 ± 0.01	31.10 ± 0.24	26.16 ± 0.02
*S. aureus*	10^–2^	TNTC	TNTC	TNTC	23.33 ± 0.16	22.30 ± 0.11	36.58 ± 0.70	26.39 ± 0.24
*S. epidermidis*	10^–2^	TNTC	TNTC	TNTC	18.72 ± 0.16	18.49 ± 0.03	32.54 ± 0.50	22.31 ± 0.08
*L. jensenii*	10^–2^	TNTC	TNTC	TNTC	21.37 ± 0.06	19.61 ± 0.21	33.84 ± 0.39	29.20 ± 0.20
*S. aureus*	10^–3^	TNTC	TNTC	TNTC	27.11 ± 0.07	25.74 ± 0.09	N/A	30.16 ± 0.19
*S. epidermidis*	10^–3^	TNTC	TNTC	TNTC	22.95 ± 0.15	22.54 ± 0.16	34.37 ± 0.87	25.33 ± 0.09
*L. jensenii*	10^–3^	TNTC	TNTC	TNTC	24.89 ± 0.23	23.42 ± 0.13	37.92 ± 1.48	33.71 ± 0.41
*S. aureus*	10^–4^	717	833	461	30.44 ± 0.19	32.22 ± 0.28	N/A	33.33 ± 0.28
*S. epidermidis*	10^–4^	446	226	364	25.90 ± 0.23	25.44 ± 0.26	N/A	29.84 ± 0.17
*L. jensenii*	10^–4^	437	326	385	28.75 ± 0.40	27.00 ± 0.04	N/A	35.89 ± 0.62
*S. aureus*	10^–5^	119	85	109	34.82 ± 0.29	34.19 ± 0.31	N/A	37.29 ± 0.16
*S. epidermidis*	10^–5^	88	38	67	30.45 ± 0.18	28.15 ± 0.13	N/A	33.37 ± 0.51
*L. jensenii*	10^–5^	33	44	44	31.01 ± 0.24	31.43 ± 0.18	37.50 ± 0.64	37.76
*S. aureus*	10^–6^	11	12	5	36.92 ± 0.57	36.3	N/A	38.35 ± 0.57
*S. epidermidis*	10^–6^	16	0	5	33.71 ± 0.61	32.12 ± 0.08	N/A	36.52 ± 0.15
*L. jensenii*	10^–6^	2	1	3	34.21 ± 0.13	35.02 ± 1.04	N/A	37.52
*S. aureus*	10^–7^	0	0	0	37.74 ± 1.61	37.41 ± 0.09	N/A	N/A
*S. epidermidis*	10^–7^	0	0	0	N/A	N/A	N/A	N/A
*L. jensenii*	10^–7^	0	0	0	38.14	36.01 ± 0.31	N/A	36.73
*S. aureus*	10^–8^	0	0	0	N/A	N/A	N/A	N/A
*S. epidermidis*	10^–8^	0	0	0	N/A	33.67 ± 1.61	N/A	N/A
*L. jensenii*	10^–8^	0	0	0	37.05 ± 0.86	37.66	37.74	N/A
*S. aureus*	10^–9^	0	0	0	N/A	N/A	N/A	N/A
*S. epidermidis*	10^–9^	0	0	0	N/A	N/A	N/A	N/A
*L. jensenii*	10^–9^	0	0	0	N/A	36.58 ± 0.18	N/A	35.88

TNTC, Too Numerous To Count. N/A: Signals below the reliable detection threshold (including sporadic or inconsistent late amplification).

### Standard curve of PMA-qPCR and CFU/reaction

**FIGURE 2:**
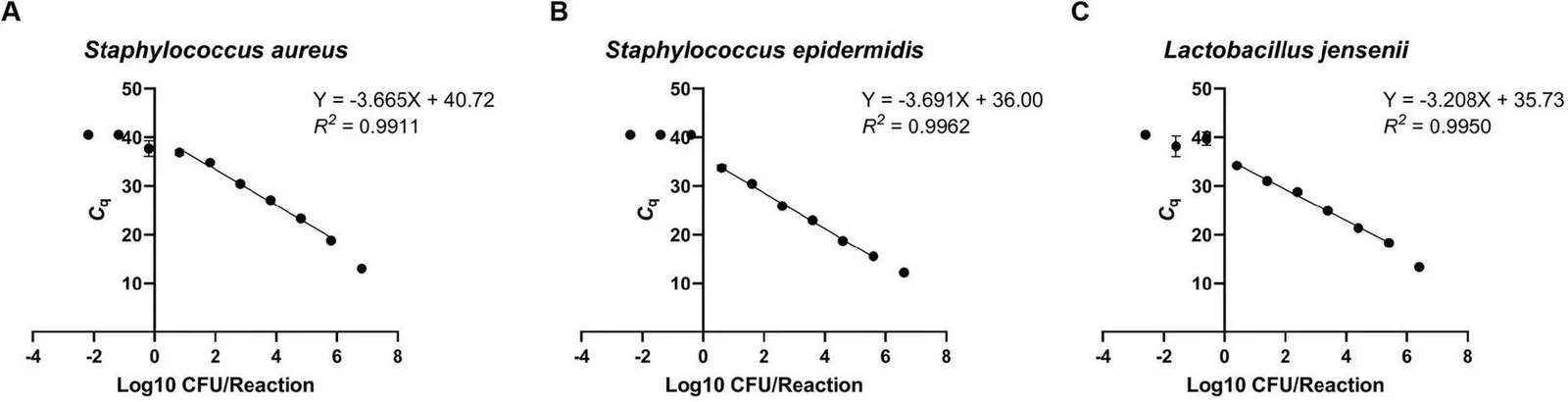
Standard curve between qPCR (*C*_q_) and Log_10_ CFU/reaction. Mean *C*_q_ values are plotted against the Log_10_ CFU/Reaction for **(A)** *S. aureus*, **(B)** *S. epidermidis*, and **(C)** *L. jensenii*. Linear regression equations and *R*^2^ values are shown. The amplification efficiency (*E*) was calculated from the slope of the regression line using the formula: *E* = 10^– 1/slope^–1.

Standard curves for each bacterium were generated to evaluate the regression coefficient (*R*^2^) and amplification efficiency (*E*) of the qPCR assay. All bacteria demonstrated high regression coefficient (*R*^2^ > 0.99) and acceptable amplification efficiency (85% < *E <* 105%) (Table 3). Specifically, *S. aureus* showed an *R*^2^ > 0.9911 with an *E* of 87.44%, *S. epidermidis* showed an *R*^2^ > 0.9962 with 98.07% efficiency, and *L. jensenii* displayed an *R*^2^ > 0.9950 with 104.98% efficiency (Figure 2).

**TABLE 3:** Linear regression parameters and amplification efficiencies of standard curve using PMA-qPCR.

Target bacteria	Slope	Y-intercept	Correlation coefficient (R^2^)	PCR efficiency (%)
*S. aureus*	−3.665	40.720	0.9911	87.44
*S. epidermidis*	−3.369	36.000	0.9962	98.07
*L. jensenii*	−3.208	35.730	0.9950	104.98

The *C*_q_ cut-off value was set at 40.5 for all bacteria (Table 4). The dilution of 10^–7^ was identified as the limit where amplification either fell below the reliable detection threshold. Consequently, the reliable limit of detection (rLOD) was determined to be the 10^–6^ dilution. The calculated rLOD values were < 6 CFU/Reaction for *S. aureus*, < 4 CFU/Reaction for *S. epidermidis*, and < 3 CFU/Reaction for *L. jensenii*. Furthermore, based on the stability of the coefficient of variation (CV), the limit of quantification (LOQ) was established at the 10^–5^ dilution. The corresponding LOQ values were approximately 65 CFU/Reaction for *S. aureus*, 40 CFU/Reaction for *S. epidermidis*, and 25 CFU/Reaction for *L. jensenii*. Assuming no DNA loss during the DNA extraction process and accounting for the elution and template volumes, reaction values were converted to volumetric concentrations. The LOQ and rLOD for each strain were calculated as follows: *S. aureus*: 5,200 CFU/mL (LOQ) and 480 CFU/mL (rLOD), *S. epidermidis*: 3,200 CFU/mL (LOQ) and 320 CFU/mL (rLOD), *L. jensenii*: 2,000 CFU/mL (LOQ) and 240 CFU/mL (rLOD).

**TABLE 4:** Determination of limit of detection (LOD) and limit of quantification (LOQ) based on *C*_q_.

Target bacteria	Dilution	Expected Log_10_ CFU/reaction	Average *C*_q_	Average observed Log_10_ CFU/reaction	Coefficient of variation (%)
*S. aureus*	10^0^	6.81	13.03 ± 0.18	–	–
	10^–1^	5.81	18.83 ± 0.17	–	–
	10^–2^	4.81	23.33 ± 0.16	–	–
	10^–3^	3.81	27.11 ± 0.07	–	–
	10^–4^	2.81	30.44 ± 0.19	2.95 ± 0.19 ( 10)	6.44
	10^–5^	1.81	34.82 ± 0.29	1.99 ± 0.27 (*n* = 10)	13.77
	10^–6^	0.81	36.92 ± 0.57	1.06 ± 0.15 (*n* = 10)	14.57
	10^–7^	−0.19	37.74 ± 1.61 (2/3)	–	–
	10^–8^	−1.19	N/A	–	–
	10^–9^	−2.19	N/A	−	−
*S. epidermidis*	10^0^	6.60	12.23 ± 0.11	−	−
	10^–1^	5.60	15.50 ± 0.16	−	−
	10^–2^	4.60	18.72 ± 0.16	−	−
	10^–3^	3.60	22.95 ± 0.15	−	−
	10^–4^	2.60	25.90 ± 0.23	2.44 ± 0.20 (*n* = 10)	8.14
	10^–5^	1.60	30.45 ± 0.18	1.51 ± 0.04 (*n* = 10)	2.81
	10^–6^	0.60	33.71 ± 0.61	0.56 ± 0.14 (*n* = 10)	24.85
	10^–7^	−0.40	N/A	−	−
	10^–8^	−1.40	N/A	−	−
	10^–9^	−2.40	N/A	−	−
*L. jensenii*	10^0^	6.40	13.37 ± 0.14	−	−
	10^–1^	5.40	18.32 ± 0.14	−	−
	10^–2^	4.40	21.37 ± 0.06	−	−
	10^–3^	3.40	24.89 ± 0.23	−	−
	10^–4^	2.40	28.75 ± 0.40	2.39 ± 0.11 (*n* = 10)	4.60
	10^–5^	1.40	31.01 ± 0.24	1.42 ± 0.04 (*n* = 10)	3.15
	10^–6^	0.40	34.21 ± 0.13	0.47 ± 0.10 (*n* = 10)	21.13
	10^–7^	−0.60	38.14 (1/3)	−	−
	10^–8^	−1.60	N/A	−	−
	10^–9^	−2.60	N/A	−	−

N/A: Signals below the reliable detection threshold (including sporadic or inconsistent late amplification).

### Comparison of PMA-qPCR and culture-based enumeration

**FIGURE 3:**
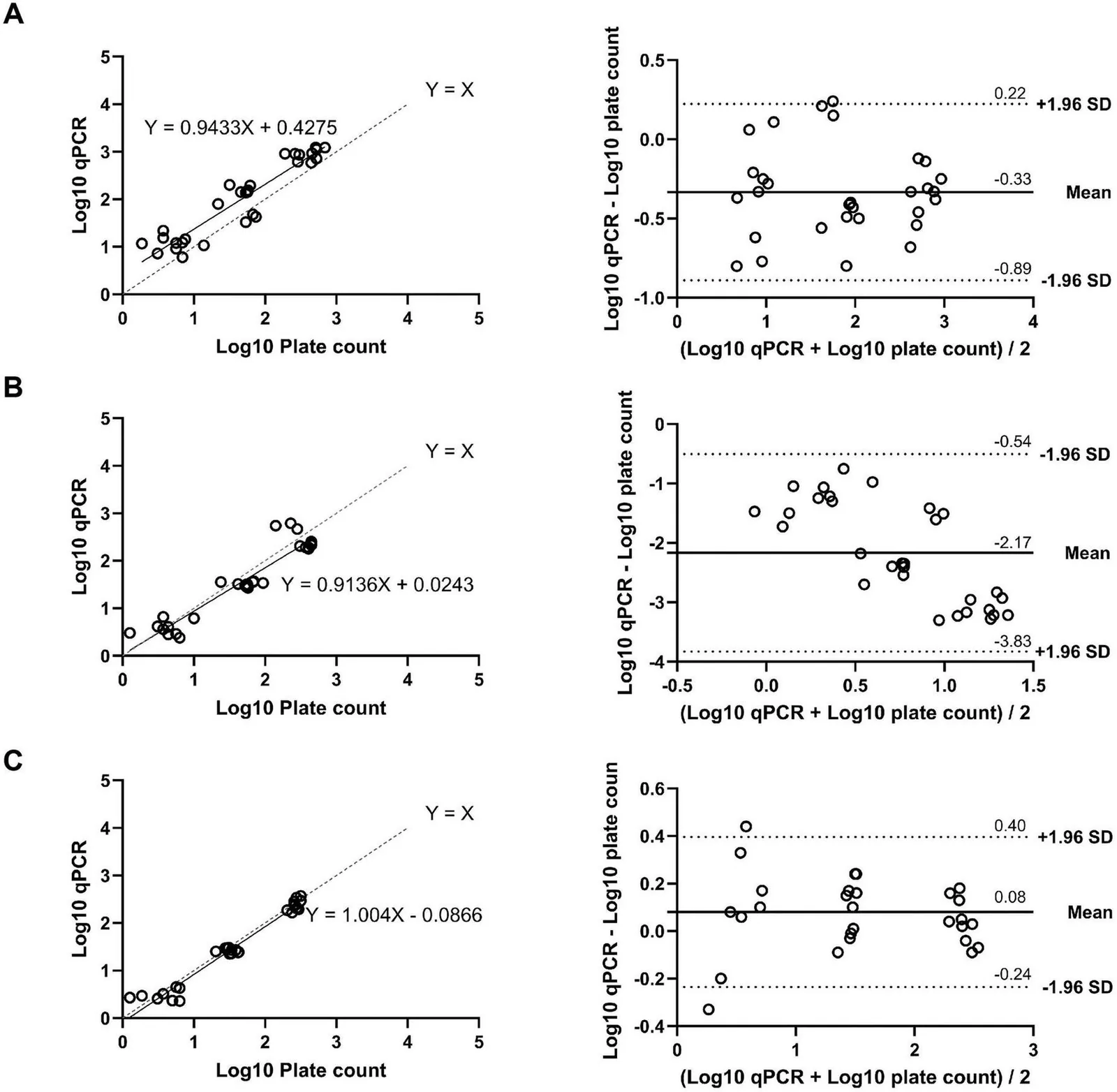
Evaluation of similarity between qPCR and plate count using linear regression and Bland-Altman analysis. Left panels represent linear regression analysis correlating Log_10_ CFU/Reaction obtained by both methods. Right panels show Bland-Altman plots assessing agreement and bias for **(A)** *S. aureus*, **(B)** *S. epidermidis*, and **(C)** *L. jensenii*. The dashed lines in Bland-Altman plots represent the 95% limits of agreement (Mean difference ± 1.96 SD).

To validate the accuracy of the established PMA-qPCR quantification model, the bacterial counts Log_10_ CFU/mL derived from the *C*_q_ values were compared with those obtained from the plate count method (Figure 3). For this analysis, data were specifically selected from the dilution range of 10^–4^ to 10^–6^, where plate counts were reliably within the countable range and *C*_q_ values remained consistent. The linear regression analysis demonstrated a strong positive correlation between the two methods for all three bacteria (Figure 3A). The regression lines for *S. aureus, S. epidermidis*, and *L. jensenii* showed slopes close to 1.0 (0.9433, 0.9136, and 1.004, respectively), indicating that the PMA-qPCR method provides enumeration results highly consistent with the plate count method across the tested dynamic range.

Furthermore, Bland-Altman analysis was performed to assess the agreement and potential bias between the two methods (Figure 3B). The differences between the Log_10_ CFU values obtained by PMA-qPCR and plate counting were plotted against the mean of the two methods. For *S. aureus* and *L. jensenii*, the mean bias was close to 0 (−0.33 and 0.08, respectively), suggesting no systematic error in the PMA-qPCR quantification. The majority of the data points fell within the 95% limits of agreement (mean difference ± 1.96 SD), indicating an acceptable level of agreement for most observations between the two methods. Although *S. epidermidis* showed a relatively larger mean bias compared to the other bacteria, demonstrating that absolute agreement is somewhat species-dependent, the plots were generally distributed within the reliable range.

### Enumeration limit of *S. aureus* in mixed-DNA background

**FIGURE 4:**
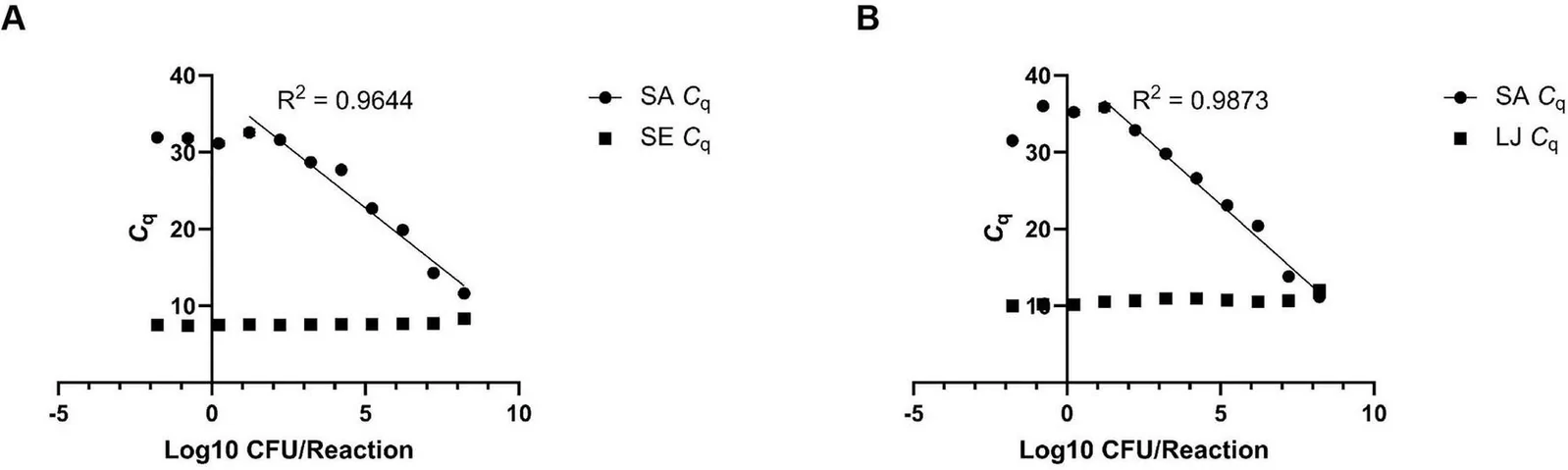
Limit of detection and quantification of *S. aureus* in a mixed-DNA background. The *C*_q_ values of *S. aureus* titrated from 10^10^ to 10^0^ CFU were measured via PMA-qPCR in the presence of a fixed high concentration (10^10^ CFU) of background bacteria. **(A)** *S. aureus* quantification in the presence of *S. epidermidis* background. **(B)** *S. aureus* quantification in the presence of *L. jensenii* background. In both panels, circular dots represent *S. aureus* and square dots represent the background bacteria. The regression line and R^2^ value indicating the linear dynamic range for *S. aureus* are shown.

Genomic DNA mixtures were prepared with fixed concentrations of *S. epidermidis* and *L. jensenii* (10^10^ CFU), while *S. aureus* was titrated in 10-fold serial dilutions ranging from 10^10^ to 10^0^ CFU. The *C*_q_ values of *S. epidermidis* (8.22 Log_10_ CFU/Reaction) and *L. jensenii* (8.22 Log_10_ CFU/Reaction) remained stable at 7.63 ± 0.23 and 10.68 ± 0.53, respectively, regardless of the *S. aureus* concentrations ranging from 10^10^ to 10^0^ CFU (Table 5 and Figure 4).

**TABLE 5:** Detection limit of *S. aureus* in the presence of high-concentration background bacteria.

Group	*S. aureus* (CFU)	*S. epidermidis* (CFU)	*S. aureus* primer (*C*_q_)	*S. epidermidis* primer (*C*_q_)
*S. aureus* + *S. epidermidis*	10^10^	10^10^	11.63 ± 0.12	8.31 ± 0.16
	10^9^		14.29 ± 0.13	7.73 ± 0.12
	10^8^		19.89 ± 0.08	7.65 ± 0.19
	10^7^		22.69 ± 0.20	7.6 ± 0.10
	10^6^		27.7 ± 0.16	7.6 ± 0.09
	10^5^		28.73 ± 0.16	7.55 ± 0.21
	10^4^		31.6 ± 0.07	7.51 ± 0.11
	10^3^		32.58 ± 0.47	7.54 ± 0.12
	10^2^		31.15 ± 0.34	7.51 ± 0.06
	10^1^		31.83 ± 0.30	7.4 ± 0.17
	10^0^		31.92 ± 0.20	7.53 ± 0.07
**Group**	***S. aureus***	***L. jensenii***	***S. aureus* primer**	***L. jensenii* primer**
*S. aureus* + *L. jensenii*	10^10^	10^10^	11.16 ± 0.11	12.06 ± 0.14
	10^9^		13.81 ± 0.11	10.65 ± 0.11
	10^8^		20.41 ± 0.09	10.51 ± 0.23
	10^7^		23.08 ± 0.04	10.74 ± 0.21
	10^6^		26.63 ± 0.13	10.99 ± 0.27
	10^5^		29.83 ± 0.17	10.96 ± 0.28
	10^4^		32.89 ± 0.22	10.65 ± 0.25
	10^3^		35.84 ± 0.52	10.54 ± 0.18
	10^2^		35.23 ± 0.37	10.15 ± 0.17
	10^1^		36.01 ± 0.20	10.21 ± 0.09
	10^0^		31.51 ± 0.11	9.99 ± 0.11

During the qPCR assays under a mixed-DNA background, the amplification specificity of each proprietary primer set was clearly verified. The post-amplification melt-curve analysis consistently yielded a single, distinct melting peak for each target without any non-specific background or cross-strain amplification (Tm ranging from 78.5 to 79.0°C for *S. aureus* and Tm = 77.0°C for *S. epidermidis*). Additionally, all no-template controls (NTC) showed no relevant signal (*C*_q_ > 40), indicating no evidence of relevant cross-reactivity under the tested conditions. Even in the presence of high concentrations of mixed-DNA background, *S. aureus* showed consistent detection, with a reliable relationship between *C*_q_ values and bacterial load from 10^10^ down to 10^3^. However, at concentrations of 10^2^ and below, the *C*_q_ values fluctuated irregularly and failed to maintain linearity, likely due to the stochastic effects at low copy numbers or background interference. Consequently, the relative limit of detection (rLOD) was determined to be the 10^3^ dilutions, corresponding to 1,000 CFU/mL. The limit of quantification (LOQ), defined by the lowest concentration with an acceptable coefficient of variation (CV), was established at 10^4^, corresponding to 10,000 CFU/mL.

## Discussion

In this study, we focused on *S. epidermidis* CICARIA, which is classified as an important skin flora in the cosmetics and probiotics industries, and optimized a viability-selective quantitative protocol by combining Propidium Monoazide (PMA) treatment with qPCR, and validated its performance. In particular, by evaluating whether pathogenic bacteria can be selectively detected not only under single-strain but also under mixed-DNA background conditions, the potential applicability to actual product quality control and microbiome research was suggested as a preliminary screening tool.

First, as a result of PMA treatment validation, in the Dead cell group, Dead^*PMA*+^ showed a higher *C*_q_ value than Dead^*PMA*–^, and in the Live cell group, Live^*PMA*+^ showed a higher *C*_q_ value than Live^*PMA*–^. These results are consistent with previous studies showing that propidium monoazide, a membrane-impermeable dye, selectively penetrates dead cells with damaged membranes, binds to DNA, and inhibits PCR amplification (Nocker et al., 2006; Yasunaga et al., 2013). In contrast, in the group not treated with PMA, the difference in *C*_q_ values between live and dead cells was minimal, reconfirming that PMA treatment can overcome the false-positive problem caused by dead-cell DNA, a limitation of conventional qPCR. This suggests that the protocol established in this study can target only live cells, excluding interference from extracellular DNA in the culture broth or dead cell DNA remaining after the sterilization process (Meng et al., 2022; Cap et al., 2025; Jansriphibul et al., 2025).

To evaluate the quantitative performance of the established PMA-qPCR method, we analyzed the correlation between *C*_q_ values and CFU counts obtained by the plate count method. The results showed high coefficients of determination (*R*^2^ > 0.99) for all three bacteria, demonstrating excellent linearity. Based on these findings, the *C*_q_ and Log_10_ CFU/Reaction regression equation serves as a useful tool for molecular biology-based estimation of viable cell counts in unknown samples. What’s interesting is that, as the dilution factor increased, some discrepancies were observed between the theoretical CFU reduction and the actual colony counts. It is presumed that some bacteria enter a Viable But Non-Culturable (VBNC) state under nutritional deficiency or physical stress, in which they maintain metabolic activity despite being non-culturable (Liu et al., 2023; Pazos-Rojas et al., 2023). While culture-based quantitative methods may underestimate bacterial counts by failing to detect VBNC cells, PMA-qPCR can potentially target membrane-intact VBNC-like cells, thereby providing a more comprehensive estimation of viable populations that may include non-culturable fractions (Elizaquível et al., 2014; Guo J. et al., 2024). However, since only qPCR analysis was performed in this study, there are methodological limitations directly measuring the metabolic activity of cells or independently verifying the complete VBNC state. Furthermore, the Log_10_ CFU/Reaction values were back-calculated based on the culture method, under the assumption that the detected DNA originates entirely from intact viable cells. Although our current regression model demonstrated excellent linearity, this approach cannot independently verify whether the quantified DNA templates are 100% derived from active live cells. Therefore, further multifaceted validation is required in future studies to strictly confirm the definitive viability of the target DNA inputs. Nevertheless, it is noteworthy that, although DNA amplification of dead cells was statistically significantly inhibited by PMA treatment, the qPCR quantification value was consistently higher than the plate count (CFU) result. In a recent study analyzing the viability of *S. aureus* in the context of skin and cosmetics (Frisch et al., 2026), the numerical gap between CFU and PMA-qPCR was similarly interpreted as indicating the formation of a potential VBNC population with intact cell membranes but a loss of culturability (Reichelt et al., 2023). In addition, the study suggested the potential for a VBNC population of Campylobacter based on the numerical difference between PMA-qPCR and plate count (CFU) in a broiler farm environment. Therefore, based on the scientific evidence of prior literature, the results of this study cautiously suggest the possibility of detecting potential VBNC cells induced by environmental stress. Future research integrating multifaceted cell viability assessments will enable more precise characterization of these unculturable bacteria.

To evaluate the agreement between the molecular quantification method developed in this study and the conventional standard method, plate counting, linear regression analysis and Bland-Altman analysis were performed (Botha et al., 2025). Regression analysis showed that the slopes of the regression lines between the two methods were close to 1.0 (*S. aureus*: 0.94, *S. epidermidis*: 0.91, *L. jensenii*: 1.00), indicating a high correlation. In addition, in the Bland-Altman analysis, the mean bias for *S. aureus* and *L. jensenii* was close to 0, and most data were within the 95% limits of agreement. Although a somewhat higher bias was observed for *S. epidermidis*, it remained within an acceptable range, suggesting that the two methods can be used complementarily. The differences in quantitative values observed in some sections are interpreted as attributable to complex factors, such as variability in DNA extraction efficiency, PCR reaction inhibition, or inherent variability in the culture method itself (Hwang et al., 2024). Consequently, this study establishes a standardized molecular framework that serves as a promising preliminary screening approach for evaluating beneficial skin isolates prior to their validation in complex cosmetic formulation matrices. However, a more critical evaluation of the Bland-Altman analysis revealed that *S. epidermidis* exhibited a relatively larger systematic bias compared to *S. aureus* and *L. jensenii*. This discrepancy suggests that while PMA-qPCR shows a high correlation with traditional plate counts, the absolute agreement remains more limited for *S. epidermidis* due to this systematic bias. The larger bias might stem from physiological heterogeneity or varying cell wall robustness among different staphylococcal backgrounds under PMA treatment. Consequently, strain-specific calibration models may be warranted to maximize quantitative accuracy for this particular species in future applications.

It is essential to eliminate interference from non-target microorganisms that may be present in the skin microbiome environment or within the product, and to selectively detect live target bacteria to establish the minimum limit of quantification (LOQ). In a recent study 2026 (Frisch et al., 2026), the PMA-qPCR method was employed to quantify the complex interactions of *S. aureus* within a skin model. However, as in that study, when conducting experiments in skin-mimicking environments, it is structurally difficult to clearly distinguish the limits of detection and quantitation (LOD/LOQ) or stain cross-interference, owing to interfering factors arising from environmental matrices. Therefore, prior to application in actual skin environments, we establish well-defined thresholds for the limits of detection and quantitation (LOD/LOQ) for each strain under standardized and variable-controlled *in vitro* multi-mix system conditions. When evaluated using genomic DNA mixtures spanning high to low concentrations (10^10^–10^0^ CFU) of *S. aureus* in the presence of high concentrations (10^10^ CFU) of background microorganisms (*S. epidermidis*, *L. jensenii*), the *C*_q_ values of background bacteria remained constant regardless of the presence of *S. aureus*, except for the 10^10^ range where the concentration of *S. aureus* was the highest. This indicates that the primers used are target-specific and that excess non-target DNA does not interfere with the PCR reaction. Additionally, the limit of detection (LOD) for *S. aureus* under this mixed-DNA background was confirmed to be approximately 1,000 CFU/mL for *S. epidermidis*, consistent with the effective detection range of this model. Specifically, the irregular *C*_q_ fluctuations and numerical inversions observed in Table 5 at concentrations below 10^3^ CFU (e.g., *S. aureus* at 10^2^–10^0^ CFU) reflect the dominance of stochastic noise and potential non-specific primer binding over true target template amplification in the presence of high background DNA ratios. Given these low-concentration variations, the limit of quantification (LOQ) for this mixed-DNA background was conservatively determined at 10^4^ CFU to ensure robust and reproducible quantification reliability for practical applications. Crucially, this operational cutoff aligns precisely with our rigorous precision threshold (CV < 20%), which was satisfied at the 10^–5^ dilution (equivalent to 10^4^ CFU) across all evaluated strains, whereas lower concentrations exceeded this margin due to technical variance. Furthermore, this determination was cross-validated by plate-count reliability; at concentrations below 10^4^ CFU (the 10^–6^ dilution), the physical colony counts dropped below the statistically valid threshold of 30 CFU per plate (Table 2). Therefore, anchoring the system-wide LOQ at this conservative level prevents sampling overdispersion and ensures strict biological consistency between our molecular data and traditional culture-based metrics. In addition, it can be seen that high detection power was demonstrated despite the combined effects of the Poisson distribution due to the low copy number and the slight signal loss from PMA treatment (Zhang et al., 2020; Ding et al., 2023; Guo et al., 2023; Botha et al., 2025; Luo et al., 2025).

Despite the robust performance of the optimized assay, several inherent methodological limitations and boundaries regarding the applicability of the PMA-qPCR method must be carefully acknowledged. First, the efficiency of PMA influx and dead cell DNA suppression is fundamentally constrained by technical artifacts. Complete suppression of dead cells remains a persistent challenge, particularly in Gram-positive bacteria due to thick peptidoglycan layers or varying physiological states of the cell membrane (Nocker et al., 2006). Furthermore, a critical limitation of this technique is the absence of a direct, independent screening method to verify the absolute penetration efficiency of PMA into compromised cells in real time. In PMA-qPCR assays evaluating target templates amid complex genetic backgrounds, it remains experimentally unverifiable whether dead cell signals are entirely eliminated, as technical challenges such as strain-specific membrane permeability differences or residual signals at low cell concentrations near the limit of detection (LOD) can introduce quantitative biases (Dinh Thanh et al., 2025; Kaur et al., 2025). Additionally, a critical limitation of the current validation framework is the exclusive reliance on autoclaved cells as the dead-cell control. While high-temperature autoclaving at 121°C was strategically employed to ensure complete membrane disruption and establish a reproducible baseline for clear LOD/LOQ determination (Frisch et al., 2026; Reichelt et al., 2023), it may not accurately replicate the physiological state of partially injured or stressed bacteria commonly encountered in cosmetic or environmental matrices (e.g., those exposed to ethanol, UV, sanitizers, or preservatives). Such sub-lethal stresses often result in structurally compromised but temporarily intact membranes, which could hinder complete PMA penetration and introduce quantitative uncertainties. Therefore, future studies should evaluate more realistic physical and chemical inactivation models to further refine the industrial applicability of this assay. Second, the quantitative accuracy of the PMA assay is highly sensitive to reaction conditions and matrix complexity. Insufficient light exposure can leave unbound dye partially non-photolyzed, potentially inhibiting viable DNA amplification, while excessively high bacterial numbers can reduce overall treatment efficiency. Moreover, fixed PMA concentrations (e.g., 50 μM) can suffer from concentration-dependent instability. Variations in dead cell density or extracellular DNA loads may induce either a Hook effect (where dead DNA is unexpectedly amplified at high concentrations) or non-specific inhibition of viable cell signals, leading to underestimation (Ding et al., 2023; Kaur et al., 2025; Wu et al., 2026). Complex formulation matrices, containing sugars, citric acid, or other organic cosmetic ingredients, may further introduce matrix interference that shields light during photoactivation or directly hinders polymerase activity. In our optimized system, however, the dead cell control consistently yielded high *C*_q_ values of 38–39, confirming that these technical interferences were minimized under our optimized, variable-controlled *in vitro* conditions. Nevertheless, given these inherent limitations, numerical discrepancies between qPCR and culture methods must be interpreted with caution, recognizing the potential boundaries of the assay before transitioning to complex commercial formulations.

In conclusion, the optimized PMA-qPCR method validated in this study offers a rapid screening alternative that complements conventional culture-based methods by providing estimates of viable cell counts, including potential VBNC populations, within a significantly reduced analysis time. Although its technical applications remain constrained by inherent limitations such as matrix effects and stochastic variations in low-concentration ranges, the standardized protocols validated herein provide a useful molecular framework. These findings demonstrate that the validated method serves as a promising preliminary screening approach with potential for future applications in skin microbiome research and the evaluation of live bacteria-based products, pending further validation in complex formulation matrices.

## Conclusion

In this study, a PMA-qPCR assay was validated for the rapid quantification of live bacteria targeting *S. aureus*, *S. epidermidis*, and *L. jensenii*, which are major strains of skin microorganisms and probiotics. This approach demonstrated excellent linearity and quantitative reliability, supported by a high coefficient of determination (*R*^2^> 0.99), and further suggested its potential to detect membrane-intact cells, which may include VBNC-like populations that are often overlooked by conventional culture methods. Furthermore, this methodology demonstrated that target strains can be selectively quantified even under a high background of co-existing non-target DNA, thereby suggesting its potential applicability to future product evaluation. In conclusion, the analytical method validated in this study is expected to serve as a useful complementary tool for preliminary screening in the cosmetics and health food industries, enhancing rather than replacing standard culture methods, and contributing to advances in skin microbiome research.

## Data Availability

The original contributions presented in the study are included in the article/Supplementary material, further inquiries can be directed to the corresponding authors.
