# Supplementary material for: Application-focused validation of a viability-selective PMA-qPCR assay for selected skin-associated and cosmetic-relevant bacteria
Source: Front Microbiol. 2026 Aug 11;17:1873833. doi: 10.3389/fmicb.2026.1873833 (PMC13504801; doi:10.3389/fmicb.2026.1873833)
Supplement: Supplementary file 1 [file Table_1.docx]

3.0kb

2.0kb

1.4kb

1.0kb

L

1

2

3

4

5

6

L

100bp

200bp

**A**

**B**

**C**

**Supplementary Figure S1. Empirical and in silico specificity validation of proprietary primer sets for *Staphylococcus aureus* and *Staphylococcus epidermidis*.**

**(A)** Agarose gel electrophoresis (1.5%) of PCR products validating primer specificity and amplicon sizes. Lane L: 100 bp DNA ladder; Lanes 1–3: *S. aureus*-specific primer system tested against (1) *S. aureus* genomic DNA (gDNA), (2) *S. epidermidis* gDNA, and (3) a non-template control (NTC); Lanes 4–6: *S. epidermidis*-specific primer system tested against (4) *S. aureus* gDNA, (5) *S. epidermidis* gDNA, and (6) an NTC. High-intensity bands are exclusively observed at the expected size (~100 bp) in lanes 1 and 5, demonstrating strict cross-species specificity and the absence of non-specific amplification or primer-dimer artifacts under a mixed DNA background. **(B)** In silico specificity validation via NCBI BLASTN analysis of the consensus sequence obtained from lane 1 (*S. aureus* amplicon), confirming definitive alignment with *S. aureus* genomes. **(C)** In silico specificity validation via NCBI BLASTN analysis of the consensus sequence obtained from lane 5 (*S. epidermidis* amplicon), confirming definitive alignment with *S. epidermidis* genomes.
